# Clinical Conundrum: Swallowing Virtual Reality as a Novel Diagnostic Tool for Severe Dysphagia after Deep Neck Infection

**DOI:** 10.1007/s00455-024-10786-z

**Published:** 2024-11-25

**Authors:** Takahiro Katsuno, Rumi Ueha, Kana Nanjo, Kazuaki Matsuda, Cathrine Miura, Taku Sato, Takao Goto, Kenji Kondo

**Affiliations:** 1 Department of Otolaryngology and Head and Neck Surgery, The University of Tokyo, Bunkyo, Japan; 2 Swallowing Center, The University of Tokyo Hospital, 7-3-1 Hongo, Bunkyo-ku, Tokyo, 113-8655 Japan; 3 Department of Otorhinolaryngology–Head and Neck Surgery, UERM Medical Center, Quezon City, Philippines

**Keywords:** Swallowing virtual reality, Deep neck infection, Dysphagia, Osteoproliferation, High-resolution pharyngeal manometry, Swallowing improvement surgery

## Case Presentation

**Fig. 1:**
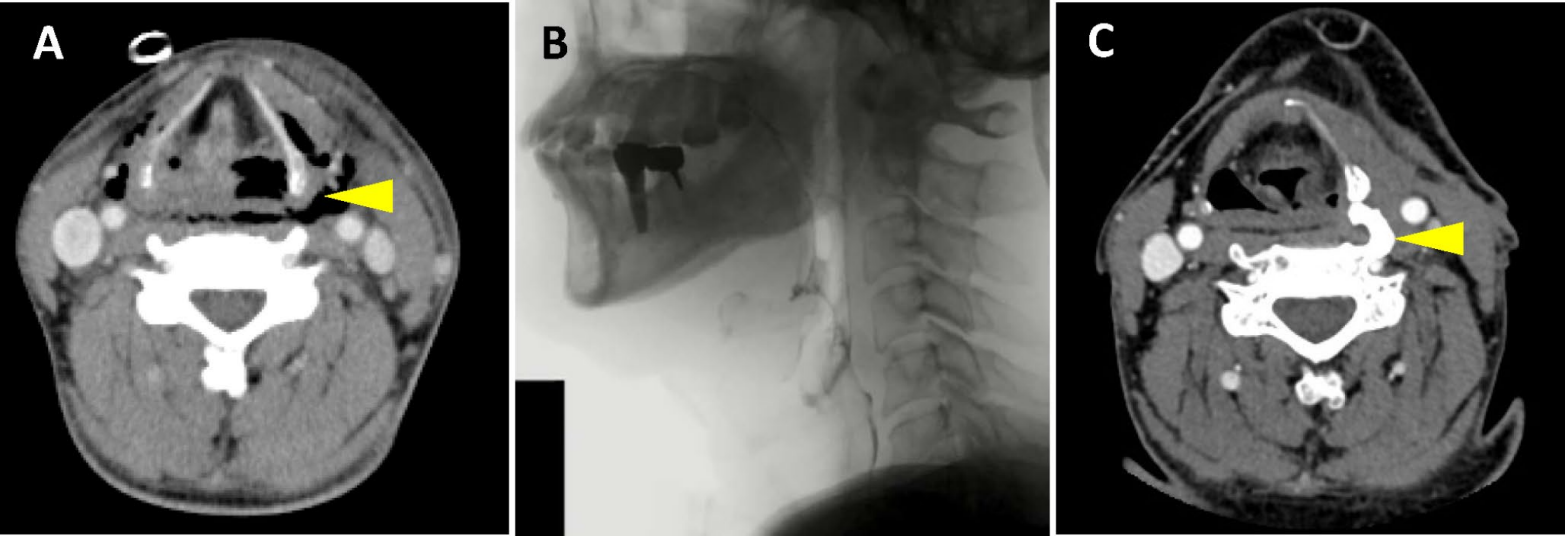
Preoperative examination. **A**: Contrast-enhanced computed tomography image of the neck at the onset of a deep neck abscess. Extensive neck emphysema was found, predominantly around the left hypopharynx (arrowhead).　**B**: Preoperative videofluorographic swallowing study. **C**: Contrast-enhanced neck computed tomography 7 months after onset revealed an abnormal bony outgrowth of the cervical vertebra (arrowhead)

A 52-year-old man was urgently hospitalized at the previous hospital because of deep neck and mediastinal abscesses (Fig. 1A) extending from the left mandible to the mediastinum, caused by caries in the left second molar tooth. He underwent abscess incision and drainage, along with tracheostomy, and received ventilator support for nearly a month. During the course of treatment, a left-sided hypopharyngeal cutaneous fistula developed, which took several months to close spontaneously. Owing to severe dysphagia, a gastrostomy tube was placed for nutritional management. He had no history of diabetes or other diseases.

Six months after the disease onset, severe dysphagia persisted, and the patient was referred to our hospital for a detailed examination of dysphagia and for treatment to improve swallowing function. Although liquid intake was possible during this time, the patient remained unable to consume solid food and was maintained on a gastrostomy tube for nutritional support. At the initial visit to our hospital, extensive neck scars were observed from the mandible to the supraclavicular area, and granulation tissue was noted around the entire circumference of the tracheostoma. Upon neck palpation, the larynx barely elevated during swallowing. Laryngoscopy revealed narrowing of the left pyriform sinus with retention of saliva. A videofluorographic swallowing study (VFSS) indicated severe impairment of laryngeal elevation with predominant right-sided pharyngeal passage and minimal aspiration (Fig. 1B). Furthermore, neck computed tomography (CT) revealed a 1.5 cm abnormal bony outgrowth originating from the anterior process of the fifth cervical vertebra, in contact with the left lateral wing of the thyroid cartilage. This bone formation was not observed previously during treatment of the abscess (Fig. 1C).

## What are the Underlying Mechanisms and Dynamics?

**Fig. 2:**
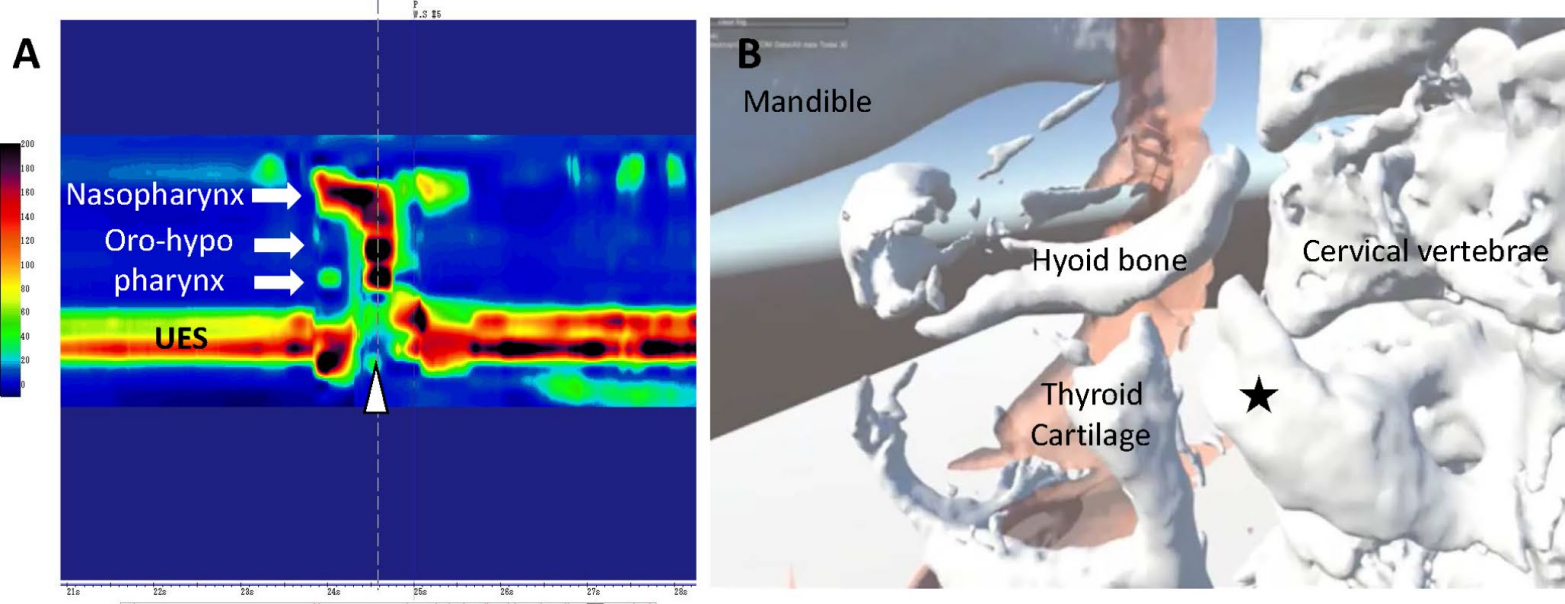
Preoperative high-resolution pharyngeal manometry and swallowing virtual reality. **A**: High-resolution pharyngeal manometry demonstrating that the upper esophageal sphincter (UES) could open during swallowing (arrowhead). **B**: Swallowing virtual reality from the lateral view detected no bony fusion between the abnormal osteoproliferation of the cervical spine (star mark) and the thyroid cartilage. Ochre indicates the pharyngeal cavity

HRM demonstrated that pharyngeal contraction pressure from the upper to lower regions was preserved, with the upper esophageal sphincter (UES) opening during swallowing (Fig. 2A). To determine the extent of adhesion around and between the bony outgrowth from the cervical vertebra and the thyroid cartilage during swallowing, dynamic swallowing CT was performed. When the dynamic swallowing CT data were rendered in virtual reality (VR) imaging (swallowing VR) [1] for assessment, a gap was detected between the cervical osteoproliferation and thyroid cartilage, enabling the thyroid cartilage to elevate slightly during swallowing (Fig. 2B, Supplemental Movie 1). Thus, although there was a strong adhesion of connective tissue between the two structures, no bony fusion was found. Furthermore, there was a significant restriction in laryngeal upward movement during swallowing, particularly on the left side, leading to compensatory swallowing primarily utilizing the right side of the pharynx.

Based on these findings, the patient was diagnosed with severe dysphagia caused by extensive neck scarring and adhesions between the reactive cervical osteoproliferation and thyroid cartilage.

### What is the Appropriate Treatment Approach?

**Fig. 3:**
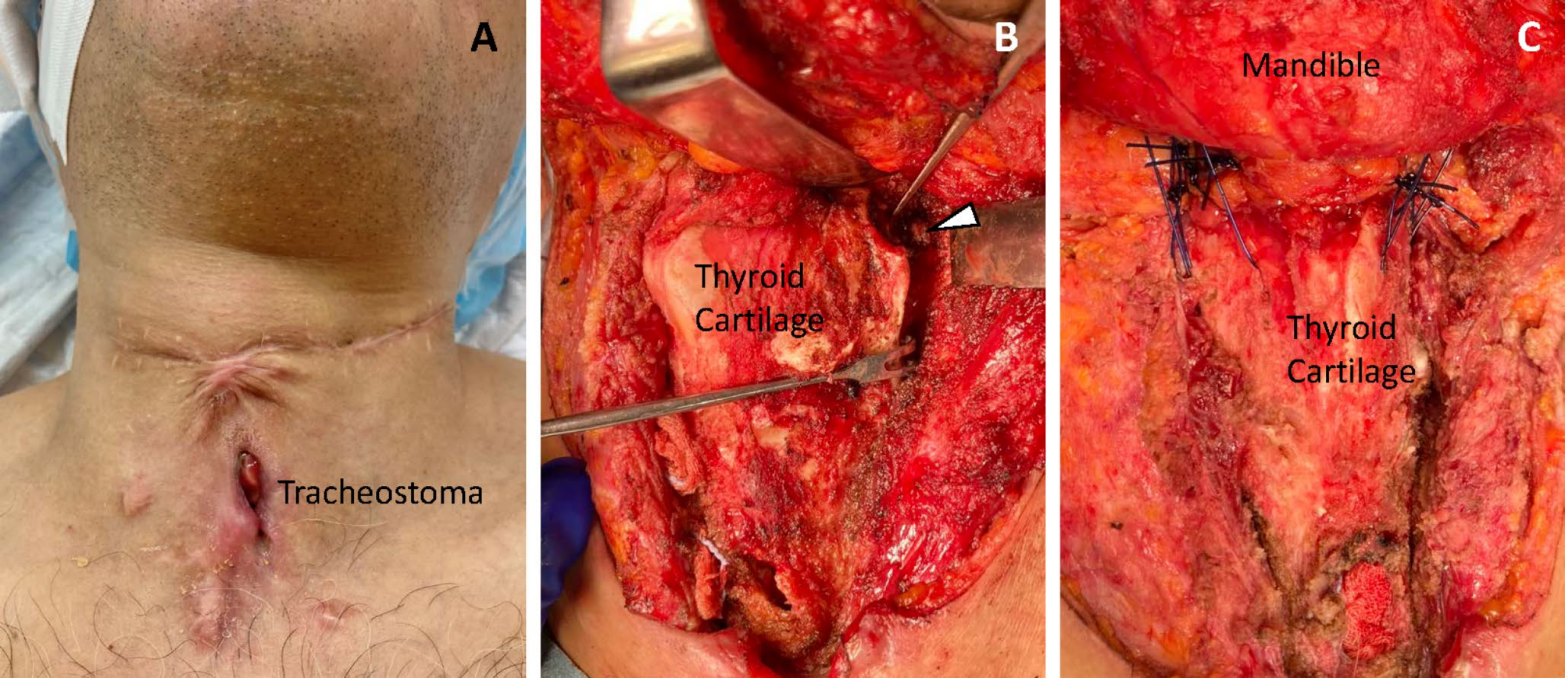
Intraoperative views. **A**: Appearance of the neck; **B**: Intraoperative view after severe scar removal and the cervical osteoproliferation site (arrowhead); **C**: View after laryngeal elevation

Conservative treatment was expected to offer minimal improvement in this patient’s swallowing function. Hence, to address the severe dysphagia, surgical interventions, including cervical scar resection, cervical proliferative bone reduction, laryngeal elevation, and tracheostoma reconstruction, were performed. Extensive scarring was present throughout the neck, causing scar dissection to take a considerable amount of time (Fig. 3A). Given the significant rigidity of scar tissue between the thyroid cartilage and abnormal cervical osteoproliferation, partial excision of the lateral thyroid cartilage was necessary to ensure adequate visualization. Subsequently, the scar was separated and a portion of the bony overgrowth was removed (Fig. 3B). Upon confirming improved mobility surrounding the thyroid cartilage, laryngeal elevation was performed by approaching the thyroid cartilage and mandible using a size 2 nylon thread with three sutures on each side (Fig. 3C).

**Fig. 4:**
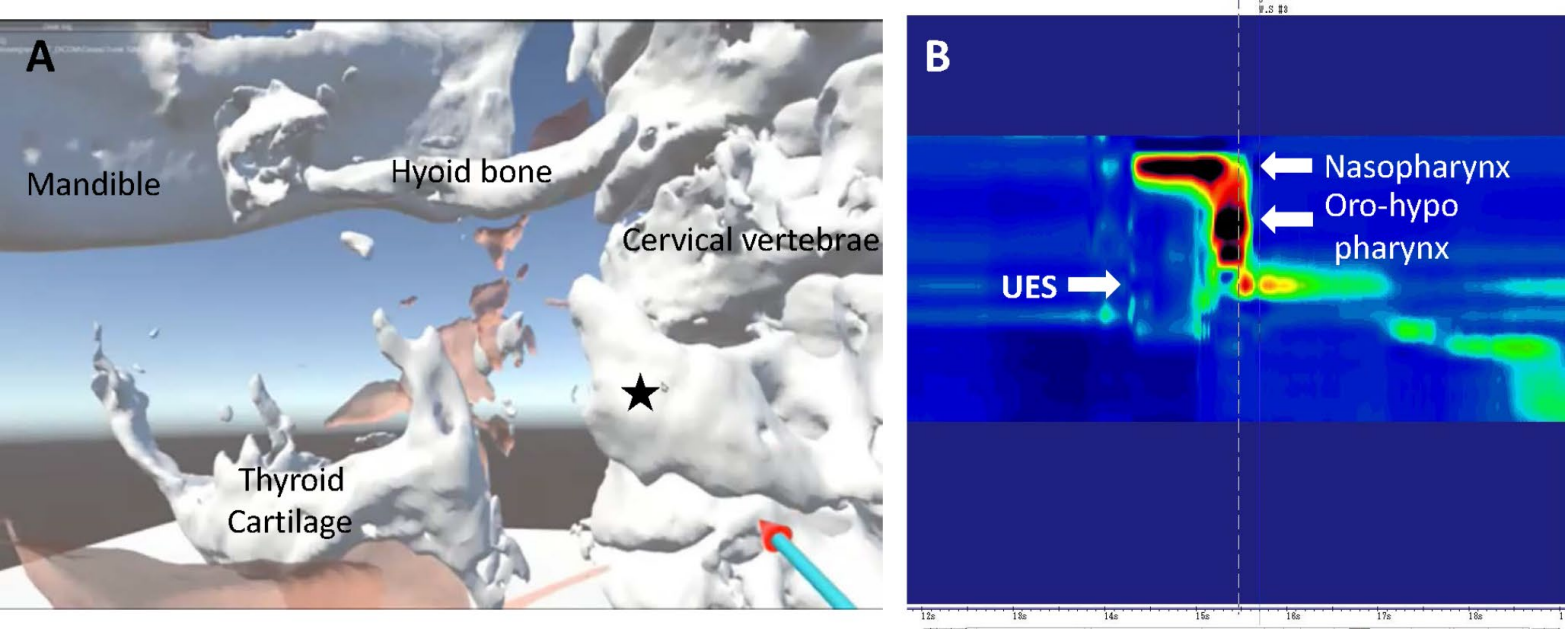
Postoperative examination. **A**: Swallowing virtual reality from lateral view. Ochre indicates the pharyngeal cavity. There was a space between the cervical osteoproliferation part (star mark) and thyroid cartilage. **B**: High-resolution pharyngeal manometry. UES: upper esophageal sphincter

Oral intake training using thickened fluids and jellies was started on the 11th postoperative day. On postoperative day 26, the patient could orally consume three meals of soft diet, leading to discontinuation of gastrostomy feeding. On postoperative day 40, swallowing VR showed an increased distance between the cervical bone proliferation and the left lateral wing of the thyroid cartilage compared to the preoperative findings, with the thyroid cartilage exhibiting satisfactory elevation during swallowing (Fig. 4A, Supplemental Movie 2). Postoperative HRM revealed a significant decrease in resting UES pressure, highlighting the effective outcome of laryngeal elevation surgery (Fig. 4B). The patient was discharged on postoperative day 41 and regained the ability to eat a normal diet by postoperative day 90, leading to the closure of the tracheostomy.

## Discussion

Deep neck infection (DNI) is a serious and potentially life-threatening condition that involves the deep fascial spaces of the neck and sometimes the mediastinum [2]. DNI, especially in the anterior cervical spaces, causes substantial scarring due to inflammation and abscesses. This scarring can impair cervical muscle function, which may lead to persistent dysphagia, even after the resolution of infection. Severe scarring may also restrict neck movements. Even with effective acute-phase treatment for DNI, scarring of the pharynx may cause complications, such as pharyngeal stenosis, laryngeal elevation impairment, and reduced pharyngeal contraction, ultimately resulting in dysphagia in approximately 20% of cases [2, 3]. Swallowing rehabilitation and other conservative treatments typically lead to improvement in swallowing in most cases; however, severe cases may require surgical intervention [4].

Osteophytes are bony outgrowths that form on vertebrae, often as a result of chronic inflammation or injury [5, 6]. The formation of anterior cervical osteophytes is a potential long-term complication, especially in severe or recurrent DNIs involving the retropharyngeal and prevertebral spaces [7]. Early and aggressive management of DNIs, including surgical drainage, antibiotic therapy, and prompt initiation of swallowing rehabilitation, is crucial for minimizing the risk of severe scarring and osteophyte formation. Regular follow-up and monitoring of these complications are recommended for patients with a history of deep neck abscesses.

When considering surgical treatment for dysphagia, an important aspect is the accurate evaluation of swallowing function using various imaging tests to identify the underlying cause and location of the impairment. Therefore, it is necessary to select an appropriate surgical procedure to address these issues. In other words, comprehensive and precise imaging examinations are pivotal for understanding the pathology of dysphagia. In the present case, VR rendering of the swallowing CT data allowed near-life-size, three-dimensional visualization, which was useful for easily understanding the movements of bony structures during swallowing, as well as identifying anatomical abnormalities and spatial changes in the pharyngeal cavity. The static imaging evaluation revealed the proximity of the abnormally proliferated cervical vertebrae and the thyroid cartilage; however, it did not allow us to observe how their positional relationship changed during swallowing movements, nor could it visualize pharyngeal contraction three-dimensionally. Moreover, as the abnormal outgrowth of the cervical vertebra was not clearly shown in VFSS, without a CT scan, we might have missed part of the pathological cause of the swallowing disorder in this patient. When a VR system is not available, swallowing dynamics can be simulated by reconstructing the swallowing CT images on a two-dimensional display. However, this image reconstruction typically requires considerable time and does not provide the same flexibility as a VR system for observing swallowing dynamics from different angles.

Swallowing VR has high usability, as it enables the visualization of swallowing dynamic images in a virtual space in approximately 3–5 min by incorporating large amounts of swallowing CT data into the system developed by the corresponding author [1].

This report presents a case of severe dysphagia resulting from extensive neck scarring and adhesion between an abnormal bony outgrowth of the cervical vertebra and thyroid cartilage following deep neck and mediastinal abscesses. To address this, we accurately assessed the condition using HRM and swallowing VR and then conducted the necessary surgical interventions, resulting in an improvement in swallowing function. In conclusion, given the complex pathophysiology of dysphagia after DNI, comprehensive evaluation using HRM and swallowing VR is useful for evaluating these patients and is anticipated to be integrated into clinical practice in the future.

## Electronic Supplementary Material

Below is the link to the electronic supplementary material.

Supplementary Material 1

Supplementary Material 2

## Data Availability

Data are available on a reasonable request.
